# Teaching undergraduate medical students Child and Adolescent Psychiatry (CAP): a Delphi study on curriculum content

**DOI:** 10.1186/s12909-018-1427-4

**Published:** 2018-12-20

**Authors:** Gill Salmon, Michal Tombs

**Affiliations:** 1 Trehafod Child and Family Clinic, Waunarlwydd Road, Cockett, Swansea, SA2 OGB UK; 2 C4ME, School of Medicine, Cardiff University, Neuadd Meirionnydd, Heath Park, Cardiff, CF14 4YS UK

**Keywords:** Child and adolescent psychiatry, Curriculum, Medical education, Learning objectives

## Abstract

**Background:**

The prevalence of psychiatric disorders in children and young people is high but despite this, many doctors have difficulty identifying and managing psychiatric disorders presenting in this age group. The purpose of this study was to determine appropriate curriculum content in Child and Adolescent Psychiatry (CAP) for a Graduate Entry Medicine (GEM) course. Doctors with a background in primary care who were also involved in undergraduate teaching rated how necessary they considered a number of knowledge, skills and attitudes items were for inclusion in the CAP curriculum.

**Methods:**

An online questionnaire study was carried out using modified Delphi methodology in two rounds. The questionnaire was derived from a list of CAP learning objectives and/or curricular content obtained from a thorough review of the literature. 23 of the 24 doctors who had agreed to participate went on to complete the round one questionnaire (95.8% response rate) with 19 also completing round 2 (82.6%). Where there was high agreement (70% or more) amongst participants, items were considered as having sufficient consensus to either accept or reject them. Mean scores were then used as a way to prioritise items.

**Results:**

At the end of round two, there was consensus to consider including 26 of the 34 knowledge items, 16 of the 20 skills items and three of the four attitudes items in the CAP curriculum. The most highly rated knowledge, skills and attitudes items were depression/ suicide; communicating with children, young people and families; and rapport building. The majority (83.3%) of round two responders, considered that the current amount of CAP teaching time was “too little”.

**Conclusions:**

Delphi methodology proved useful for determining consensus and the priority rankings of the CAP knowledge, skills and attitudes items can now be used to help educators determine which topics to focus upon. The study findings support the need for additional CAP teaching time in the GEM curriculum and will help to shape new CAP content. Additional formal CAP teaching time has already been incorporated into the psychiatry speciality attachment, a new clinical skills session has been developed and CAP topics have been introduced into written and clinical examinations.

## Background

It has been shown that the prevalence of psychiatric disorders in Children  and Young People (CYP) is high, for example 8% of girls and 11% of boys aged 5-15 years in one large UK study [1]. Of note, whilst only a quarter of the CYP in that study with a psychiatric disorder had been seen in a specialist health service in the previous year, nearly a half had had contact with their General Practitioner (GP) and they were also more likely than children with no disorder to present in other health settings such as Accident and Emergency or outpatient departments [1]. It is therefore concerning that GPs as well as other specialists e.g. paediatricians, have been reported to have difficulty identifying and managing psychiatric disorders presenting in CYP [2–4] and that newly qualified GPs feel unprepared, particularly in relation to CAP [5]. Is has been suggested that the care of CYP with psychiatric problems could be improved by adapting undergraduate education to reflect the future needs of doctors working in primary care [6]. These findings support the need for all medical students to have a foundation in Child and Adolescent Psychiatry (CAP) regardless of their future career choices [7, 8].

A number of studies have been conducted over the years in the UK [9, 10], Europe [11, 12], Australia [13], and USA and Canada [14–16], examining CAP teaching offered to undergraduate medical students. Despite this, there is still no clear consensus on the optimum number of hours CAP teaching that medical students may require before they graduate nor which topics should be taught [17]. What these studies do have in common however is that many recommend that the undergraduate CAP curriculum should be made more relevant to students given the majority are not planning to become psychiatrists. One UK study, for example, has shown that nearly 25% of medical students are considering general practice as a career [18]. This may increase to 50% in the future given recent political pressures in the UK to increase the numbers of students going into primary care [19]. Similar conclusions are also made in a CAP curriculum review in a Canadian medical school [20] as well in several other studies examining which topics should be included in a CAP curriculum [6, 13, 21].

Studies of undergraduate curriculum content in other specialties e.g. urology [22, 23] as well as psoriasis and psychiatry [24–26] have also concluded that the teaching offered to medical students needs to equip them with the knowledge and skills required in primary care careers. The authors of these studies have sought the opinions of non specialists, taking the view that they are in a better position than specialists to know what should be included in an undergraduate medical curriculum. These findings informed the aims and methodology of this study, which used Delphi methodology to determine what a panel of non specialists thought should be included in an undergraduate CAP curriculum to address the lack of consensus in this area. The study took place at a time when the undergraduate medical curriculum as a whole in the Swansea Graduate Entry Medical (GEM) School was under review. This provided an opportunity to reflect upon the CAP curriculum and to explore the educational needs of GEM students in relation to Child and Adolescent Psychiatry (CAP). The findings informed decision making around teaching content and were used to influence decisions about future CAP teaching and curriculum at the School. The aim of the study was therefore to develop consensus among doctors with a background in primary care who were also involved in undergraduate teaching as to how necessary they considered a number of knowledge, skills and attitudes items were for inclusion in the CAP curriculum.

## Methods

The Delphi technique enables the views of people, who are considered to be experts in their field, to be sought and combined without them having to meet. Rather than a simple survey, it could be seen as a virtual meeting or a process of group decision making [27, 28]. It has been shown repeatedly that if expert judgment is required to answer questions, then the averages obtained from a group decision making process such as the Delphi are superior to those obtained from individuals [27].

Delphi methodology has been used to determine consensus on educational needs and curriculum planning in a range of healthcare environments both at postgraduate [29, 30] and undergraduate level [24, 26, 31, 32]. It is common place, and acceptable practice, to use a modified version of the Delphi technique whereby the questionnaire that is developed for circulation to the participants is based on a list of issues obtained from a thorough review of the literature. This ‘confirmatory’ approach to round one was used by Alahlafi and Burge in their modified Delphi study, which aimed to identify suitable content for an undergraduate psoriasis curriculum [24] and Walley and Webb [32] in their modified Delphi study aiming to develop a core undergraduate curriculum in clinical pharmacology and therapeutics and is the one taken in this study. A questionnaire was developed based on a literature review using key words and phrases including: medical students/ medical education/ curriculum/ college/ students/ child and adolescent psychiatry/child psychiatry and adolescent psychiatry. Databases searched included: PsychInfo (363); Embase (81); Ovid Medline (31); and PubMed (175). Against each database, the number of potentially relevant papers generated by the search is shown. Relevant papers found were also searched in order to identify additional studies. The final questionnaire was derived from an analysis of 22 relevant papers.

CAP learning objectives and/or curricular content identified from these papers were listed under the categories of knowledge, skills and attitudes. A pilot questionnaire was developed that included 33 knowledge (e.g. conduct disorders, psychotic disorders, medication), 20 skills (e.g. interviewing CYP, assessing mental state) and four attitudes items (e.g. building rapport). Participants were asked to rate their view of how necessary they considered the item to be for inclusion on an undergraduate CAP curriculum using a 5-point likert-type scale (i.e. 1 = unnecessary, 2 = unimportant, 3 = worth considering, 4 = important, 5 = definitely necessary). Participants were also asked whether they thought the current amount of formal CAP teaching on the course (estimated at less than 6 h) was “too much”, “about right” or “too little”. Participants were invited to make free text comments in a space after each item and were also asked an additional open ended question at the end of the survey “*Please list any additional knowledge, skills and attitudes which you think should be included on an undergraduate medical curriculum for child and adolescent psychiatry in the space below*”.

Bristol Online Survey (BOS) was chosen to host the Delphi questionnaire as this offered many advantages including: speed of data collection and analysis; lower cost; data held securely; better quality data collection as well as being able to be in direct communication with participants [33] and it has already been used in modified Delphi studies relating to health care [34, 35]. Seven participants agreed to take part in the pilot study and completed the pilot questionnaire. As a result of feedback, the wording of the instructions at the start of the online survey was altered slightly for the round one and round two surveys to “*please feel free to make any additional comments you would like to make in the free text space adjacent to each item. You can also use this space if you have suggestions for rewording the question if you think it is unclear or if you wish to make additional comments e.g. about why you chose this rating of priority*”.

After the pilot study, there was a ‘recruitment round’ whereby 79 potential participants including 67 GPs offering Community Based Learning (CBL) placements and 12 doctors working within the medical school, were approached with an email of invitation and Participant Information Sheet, which informed that the Delphi study was being conducted to gain a consensus on potential learning objectives for GEM students in CAP as perceived by non psychiatrists. Participants were also made aware that the study results would form part of a wider educational needs assessment for undergraduate teaching in CAP, a summary of which would be made available to the GEM School in Swansea. The inclusion criteria for the study were: GP by training; clinically working (or have worked) in a setting where CYP are regularly seen; scope of work involves undergraduate medical education; willing to participate.

Twenty-four agreed to participate, comprising 15 GPs offering CBL placements and nine doctors working in the medical school (i.e. 30.4% take up rate). These were sent an email with a link to the BOS questionnaire. Reminder emails were sent after approximately 10 days to those who had not responded, with a further reminder a few days before the survey closed (three weeks after opening). Twenty three of the 24, completed the round one online questionnaire (95.8% response rate) of whom 13 were male and 10 were female. Their mean age was 46.35 years (standard deviation = 9.51) with a mean number of years post qualification of 22 (standard deviation = 8.85).

An additional knowledge question about CAP self-help books and resources was proposed by two of the participants in round one and was added to the round two questionnaire before a link was then sent by email to the 23 responders. Participants were also given information about each item’s rating in the first round i.e. the percentage of participants who had scored each item as either “important” or “definitely necessary” to be included in the CAP curriculum. Participants were then asked to re-rate each item. Reminders were sent out by email after 10 days and again three days before the round two survey closed (three weeks after starting).

## Results

Consensus was considered to have been achieved to place an item on the CAP curriculum after round two if at least 70% of participants rated it as ‘important’ or ‘definitely necessary’. Consensus not to place an item on the CAP curriculum was considered to have been achieved if at least 70% of participants rated the item as as ‘unnecessary’ or ‘unimportant’ [34]. Given the number of items and pressure on available curriculum time, a statistical approach was then used to determine which of the CAP topics considered “important” or “definitely necessary” should be prioritised for inclusion in the CAP curriculum. This was based on mean scores and standard deviations calculated using Excel after the second round questionnaire [6].

### Amount of CAP teaching time

Sixteen of the round one Delphi respondents (69.6%) thought that the current amount of formal CAP teaching time was “too little” and seven (30.4%) responded it was “about right”. This compares with 15 of the round two respondents (83.3%) who thought that the current amount of formal CAP teaching time was “too little” and three (16.7%) who responded it was “about right”. In both rounds, no participants thought it was “too much”. Textual comments provided by participants such as “*I am not sure how this compares with other specialties but it sounds too little”* and *“… much of what I have learnt about child psychiatry has been in the post graduate setting of GP”* further demonstrate participants’ perceptions with regards to current teaching of CAP.

### CAP knowledge

At the end of both rounds, consensus had been reached for 26 of the knowledge items to be included in the CAP curriculum (see Table 1). Participants provided free text comments that demonstrate the significance of CAP in their role as GPs. One participant commented, *“Child and adolescent psychiatry forms a significant part of the workload of a GP. Learning to accurately assess, diagnose and appropriately manage conditions in primary care is important”.* The extent to which GPs felt that CAP is an important knowledge area was reflected in more specific comments to conditions such as depression *“very common in GP”* and *“Underdiagnosed and a cause of significant harm to patients and their families”;* and for anxiety disorders *“Such a common confounding presentation in GP”.*

**Table 1:** CAP knowledge to be included on GEM curriculum

Rank after R2 based On mean score	Knowledge items	% scoring “important” or “definitely necessary”		Mean after R2	Std Dev R2
		R1	R2		
1	Depression and suicide	96%	100%	4.95	.23
2	Sexual abuse	96%	100%	4.89	.32
3	Physical abuse	95%	100%	4.84	.37
4	Normal emotional, social, and cognitive development	96%	100%	4.79	.42
5	Eating disorders	100%	100%	4.74	.45
6	Non suicidal self harm	91%	100%	4.68	.48
7	Anxiety disorders	87%	100%	4.58	.51
8	Bullying/cyberbullying	95%	95%	4.53	.61
9	Substance misuse	83%	100%	4.47	.51
10	Overweight/obesity	77%	90%	4.42	.69
11	Family interactions and their relevance	83%	95%	4.42	.61
12	Psychotic Disorders/schizophrenia	83%	95%	4.37	.60
13	ADHD	91%	95%	4.32	.58
14	Autism Spectrum Disorders	87%	100%	4.32	.48
15	The effects of mentally and physically ill parents on children	87%	95%	4.32	.58
16	The impact of a child’s illness on the family	74%	95%	4.32	.58
17	The emotional problems of physically ill children	87%	95%	4.32	.58
18	The interaction between psychosocial and physical factors in paediatric disease	83%	90%	4.32	.67
19	Which psychological problems in CYP can be managed by primary care/other agencies and which to refer to specialist CAMHS	83%	79%	4.32	.82
20	The range of services available to help CYP with mental health problems	77%	90%	4.26	.65
21	Medications used to treat common disorders such as ADHD and depression	78%	89%	4.22	.65
22	Diagnosis of learning difficulties	70%	84%	4.21	.71
23	Parenting issues e.g. separation, divorce, abuse	83%	95%	4.21	.54
24	The treatment options available for CYP with psychiatric disorders	83%	95%	4.16	.50
25	How to refer to specialist CAMHS	70%	79%	4.00	.82
26	Predisposing, precipitating and maintaining factors that influencing aetiology, course or compliance with treatment of disorders	74%	74%	3.89	.66
27	Psychogenic abdominal pain	63%	58%	3.79	.79
28	Self help books/resources about CYP’S mental health to recommend	n/a new Q for R2	63%	3.79	1.13
29	Somatoform disorders	52%	53%	3.63	.96
30	School refusal	48%	42%	3.53	.70
31	Conduct disorders	61%	47%	3.42	.90
32	Infant regulatory disorders e.g. excess crying, feeding and sleeping difficulties	44%	37%	3.42	.90
33	Attachment disorders	52%	32%	3.37	.76
34	Tic disorders	43%	37%	3.11	.99

As can be seen in Table 1, eight knowledge items did not reach the required statistical level for consensus. For these items, textual comments revealed that some participants did perceive certain areas to be important for the work of a GP. For example, for regulatory disorders during infancy participants commented that it is “*Very common and a real issue with parents”* and “*Often managed by the health visitor but GPs need to have knowledge and be able to diagnose the serious from the normal”.* Similarly, for tic disorders participants commented that *“GPs commonly see tics and aside from major tic disorders, the local provision of specialist services is patchy”* and *“GPs need to be more aware. Diagnosis is relatively easy and it will be managed in secondary care”.* Another knowledge items that did not reach consensus was psychogenic abdominal pain. Again, textual comments suggested that some participants perceived it to be important commenting “*Also very common in GP”* and *“The GP will often have patients with this problem and by being taught how to manage it properly it will reduce referrals and improve diagnosis in general practice”.*

The top ten ranked CAP knowledge items based on their mean score in round two were as follows: depression and suicide; sexual abuse; physical abuse; normal social, cognitive and emotional child and adolescent development; eating disorders; non suicidal self-harm; anxiety disorders; bullying /cyberbullying; adolescent substance misuse and overweight/obesity.

### CAP skills

At the end of both rounds consensus had been reached for 16 of the 20 skills items (see Table 2). Free text comments provided additional insights into what it is that GPs perceive to be of particular importance and why. For example, one participant commented, *“We are increasingly seeing child and adolescent issues in general practice. As we are the first port of call, these skills need to be present and not just in a specialist service”.* This was further reinforced with comments made to more specific skills such as interviewing parents about their children and taking a CAP history “*I cannot remember learning how to do this in medical school. All that I have learnt is through self directed learning or through experience”.*

**Table 2:** CAP skills to be included on GEM curriculum

Rank R2	Skill items	% scoring “important” or “definitely necessary”		Mean R2	SD R2
		R1	R2		
1	Communicate with CYP/ families in a developmentally appropriate way	96%	100%	4.84	.37
2	Assess suicidality	100%	100%	4.84	.37
3	Assess suspected sexual/physical abuse	100%	100%	4.79	.42
4	Interview parents and take a Child and Adolescent Psychiatric history	96%	95%	4.68	.58
5	Assess mental state in CYP	96%	100%	4.68	.48
6	Interview children	100%	95%	4.63	.60
7	Interview adolescents	100%	95%	4.63	.60
8	Differentiate between normal and pathological behaviour in CYP	96%	95%	4.61	.61
9	Make a formulation, consider differential diagnoses and treatment	96%	95%	4.58	.61
10	Recognize/assess common mental health problems in CYP	87%	95%	4.47	.61
11	Interview families	83%	90%	4.32	.67
12	Break bad news	77%	89%	4.28	.67
13	Manage agitated patients	78%	79%	4.11	.74
14	Manage common behaviour problems	78%	83%	4.06	.80
15	Accurately screen CYP for mental health problems	83%	74%	3.95	.97
16	Recognize factors that may be influencing the presentation, course or compliance with treatment	83%	84%	3.95	.52
17	Make a referral to specialist CAMHS/explain CYP and families	61%	53%	3.63	1.12
18	Monitor medication in accordance with evidence based guidelines	57%	37%	3.11	.99
19	Deliver basic/supportive psychotherapy	44%	32%	3.05	.97
20	Start medication in accordance with evidence based guidance	51%	21%	2.89	.94

Examination of textual comments made to the four items for which there was no consensus suggest that some participants did not feel they have the time or resources to provide a service. For example, in response to the item on delivering basic/supportive psychotherapy, participants wrote “*Scope for GPs to deliver this is limited”* and *“More secondary care -we only get 10 minutes!!!!”.* Indeed, textual comments suggested that some participants perceived the role of the GP to be one of signposting, and this was reflected in responses made to making a referral to specialist CAMHS and explaining the process to children and families. Participants wrote “*Maybe comes more with experience as opposed to needing early training”.* Similarly, for the item on starting and monitoring medication for child and adolescent mental health disorders participants commented “*I do not think that GPs should be starting medication for psychiatric problems in children, only under the supervision of a specialist*” and “*That really is secondary care, a more specialist role*”.

The top ten ranked CAP skills items based on their mean mean score in round two were as follows: communicate with CYP and families in a developmentally appropriate way; assess suicidality; assess suspected sexual/physical abuse; interview parents and take a CAP history; assess mental state in CYP; interview children; interview adolescents; differentiate between normal and pathological behaviour in CYP; make a formulation and consider differential diagnoses and treatment; and recognize and assess common child or adolescent mental health problems.

### CAP attitudes

At the end of round one, consensus had been reached for all four of the attitudes items to be included in the CAP curriculum (see Table 3). This had reduced by the end of round two, to three.

**Table 3:** CAP attitudes to be included on GEM curriculum

Rank R2	Skill items	% scoring ‘important’ or ‘definitely necessary’		Mean R2	SD R2
		R1	R2		
1	Vary the style of communication skills used in order to develop a rapport with a child/family	91%	95%	4.63	.60
2	Recognise that social and psychological factors interact/affect emotional/ physical development	87%	100%	4.37	.50
3	Consider and assess for psychological factors regardless of the nature of CYP’s illness given their high prevalence	70%	74%	3.95	.71
4	Promote awareness of the emotional needs of CYP and families	74%	69%	3.79	.63

## Discussion

The purpose of the study was to determine appropriate content for an undergraduate Child and Adolescent Psychiatry (CAP) Curriculum in Swansea Graduate Entry Medical (GEM) School. Doctors with a GP background who were also involved in undergraduate teaching were asked to participate in a modified Delphi Study as they were considered to be in the best position to know which CAP topics should be included.

Participants in the Delphi study considered that the total time currently teaching CAP topics in Swansea GEM School is too little. This is unsurprising given the current allocated CAP teaching time is considerably lower than the average of 20 h CAP teaching time reported in surveys of other medical schools in the UK [10] and in Europe, Australasia and North America [17]. Given that CYP commonly experience psychiatric disorders [1], and the increased availability of effective interventions [36], Sawyer et al. [17] recommend that *“higher priority to be given to child and adolescent psychiatry teaching in medical schools to reflect the size of the public health problem posed by child and adolescent mental disorders in many communities” (p145).*

At the end of round two, there was consensus to consider including 26 knowledge, 16 skills and three attitudes items in the new CAP curriculum. For the items where no consensus was achieved, free text comments made by respondents were examined to make a final decision on whether to consider including the item in the CAP curriculum [34]. For the eight CAP knowledge items for which there was no consensus to include or exclude, the free text comments made by respondents suggested that a further three of these i.e. psychogenic abdominal pain, regulatory disorders and tics were important and/or common problems encountered in primary care and should be retained for consideration. In contrast, for the four CAP skills items and one CAP attitude item for which there was no consensus to include or exclude, the free text comments made by respondents did not suggest that any of these items should be considered for inclusion in the curriculum. For these skills items, e.g. making a referral to specialist CAMHS, starting or monitoring psychotropic medication, and deliver basic/ supportive psychotherapy, the free text comments in the main cited lack of time in primary care or the item being in the domain of secondary care.

Mean scores in round two were then used to prioritize items. Three of the five highest ranking knowledge items i.e. depression (ranking first) and normal social, cognitive and emotional development and eating disorders (ranking fourth and fifth respectively) have also ranked highly in other studies [6, 12, 13]. Emotional disorders including depression (ranking first) and anxiety (ranking seventh) have a prevalence rate of 5.6% in 11–15 year olds in the UK [1] This supports the priority rankings of these disorders in this study. In one large UK study, it was reported that nearly half of the CYP with a mental disorder had seen been seen in primary care at some point during the previous year [1].

The five highest ranking skills were also similar to those found by other authors e.g. communicating with CYP and families in a developmentally appropriate way [6, 11, 21, 37], assessing suicidality and mental state [6] and assessing/diagnosing child abuse [21]. Other studies have also ranked ‘clinical assessment’/ ‘assessment of families’/and ‘assessment of psychopathology’ highly [11–13].

Reassuringly, given that the prevalence of depression has been estimated to be 2–8% amongst adolescents [38], the top ranked knowledge item in this study was ‘depression and suicide’ and the second ranked skills item was ‘assess suicidality’. Both were also seen as a priority in Sawyer et al’s [17] review of undergraduate CAP teaching which states graduating medical students “*must be able to have the ability to accurately assess and provide acute management for adolescents with depression and suicidal ideation” (p.144).*

When considering low ranking CAP items in their study, Lempp et al. [6] commented “*against the background of the ongoing discussion of over-prescribing, risks and adverse effects of psychotropic prescriptions in children and adolescents (e.g. SSRI and suicidality), it is rather surprising that neither the item “starting psychopharmacology” nor “continuing/monitoring psychopharmacotherapy” was rated as important” (p.447)*. Of interest, both of these items were also low ranking in this study, with free text comments suggesting this is seen as a specialist role.

The study findings supported the need for additional CAP teaching time in the GEM curriculum and have already shaped new CAP content. An example of this has been the introduction of a new clinical skills session focussing on interviewing a depressed adolescent using a simulated patient as well as the expansion of the CAP teaching for students on their psychiatric placement from a half day to a whole day. The study findings have also been disseminated to psychiatric and paediatric teaching colleagues as well as curriculum programme planners in the Swansea GEM programme to facilitate discussions as to how CAP teaching and topics can be integrated into the teaching arranged by other specialties. In addition, CAP topics have been introduced into written and clinical examinations [10].

Given the low number of CAP teaching hours currently offered, and the high number of knowledge, skills and attitudes items that Delphi participants in this study consider should be included in the curriculum, focussing on the rankings of items will help educators in Swansea GEM school prioritise what to include. Previous studies have shown that it is possible to make teaching seem more relevant to the majority of students by prioritising common clinical presentations e.g. depression and ADHD, and focussing on the teaching of CAP skills and attitudes rather than CAP knowledge [6, 21, 37]. Greater collaboration between child and adolescent psychiatrists, psychiatrists and paediatricians [9, 15, 39] should also be considered, recognising there can be overlap as to which topics belong in which curriculum as e.g. safeguarding, depression and Autism Spectrum Disorders. Offering more CAP teaching in primary care settings is also worth considering [6].

Modified Delphi methodology was appropriate to use to determine which CAP topics should be in the curriculum and which should be prioritised. Response rates were well above Sumsion’s [40] recommended 70% required to maintain a Delphi study’s rigour. The use of modified Delphi methodology avoided the need for a first round to ‘explore’ issues as well as having to have a questionnaire that was too brief, which other studies have found to be a limitation [12, 16]. Terminating after only two rounds of questionnaires reduced the risk of drop out [41, 42] and by using an online survey such as BOS, the usually long Delphi process [41] was speeded up considerably. Notably both of the statistical methods used yielded similar results.

Nevertheless, this study is not without some limitations and these should be recognised. Although measures were put into place to overcome the weakness of the survey approach and to mitigate potential bias, a more extensive qualitative round with subject experts to complement the literature review may have strengthened the study's findings.  In addition, the Delphi participants all had a general practice background. A panel which included a more diverse selection of stakeholders, for example child and adolescent psychiatrists receiving referrals, might have added useful information and aided the translation of the results into the local setting. Additional insights may have been gained if, as well asking participants for their views about the amount of formal CAP teaching on the curriculum, those replying that it was “too little” had also been asked, what could be reduced to make room for more CAP. The version of the online tool (BOS) used did not allow for the feedback of textual data to participants which may have inhibited them from understanding each other’s opinions. It is worth noting that the option to make free text comments was not taken up by many participants. Moreover, it is important to note that the study findings are limited by it being conducted in only one institution and therefore cannot be generalised to another context.

## Conclusion

Despite the study’s limitations, the findings provide some valuable insights at a local level which proved useful for CAP curriculum planning in Swansea and the methodology used recognises that most students will find themselves in primary care careers [19]. Further research may be needed however before recommendations for national and international standards for CAP teaching can be developed [6, 11, 13, 17]. This could involve a broader sample of stakeholders including patients as well as specialists and non specialists [24] and/or combining results from multiple medical schools.

## Abbreviations

CAP: Child and Adolescent Psychiatry
CBL: Community Based Learning
CYP: Children and Young People
GP: General Practitioner
